# Clinical, Radiological, and Pathological Investigation of Asbestosis

**DOI:** 10.3390/ijerph8030899

**Published:** 2011-03-22

**Authors:** Takumi Kishimoto, Katsuya Kato, Hiroaki Arakawa, Kazuto Ashizawa, Kouki Inai, Yukio Takeshima

**Affiliations:** 1 Asbestos Research Center, Okayama Rosai Hospital, 1-10-25 Chikkomidorimachi,Minamiku Okayama City, 702-8055, Japan; 2 Department of Radiology, Okayama University School of Medicine, 2-5-1,Shikata-cho, Kitaku,Okayama City, 702-8558, Japan; E-Mail: kato-rad@cc.okayama-u.ac.jp; 3 Department of Radiology, Dokkyo Medical University, 880, Kitakobayashi, Mibumati, Shimotuga-gun, Tochigi City, 321-0293, Japan; E-Mail: arakawa@dokkyomed.ac.jp; 4 Department of Radiology, Nagasaki University School of Medicine, 1-7-1 Sakamoto,Nagasaki City, 852-8501, Japan; E-Mail: ashi@net.nagasaki-u.ac.jp; 5 Department of Pathology, Hiroshima University School of Medicine, 1-2-3 Kasumi, Minamiku, Hiroshima City, 734-0037, Japan; E-Mails: koinai@hiroshima-u.ac.jp (K.I.); ykotake@hiroshima-u.ac.jp (Y.T.)

**Keywords:** asbestosis, peribronchiolar fibrosis, IPF/UIF, asbestos body

## Abstract

By the radiological examination, differential diagnosis of asbestosis from chronic interstitial pneumonia such as IPF/UIP is difficult. The pathological features of asbestosis show the peribronchiolar fibrosis which suggest that asbestos fibers cause the inflammation of bronchioli. Therefore, the criteria for pathological diagnosis of asbestosis in 2010, contain the finding of peribronchiolar fibrosis again. Chest CT scanning including HRCT for total of 38 cases clinically diagnosed asbestosis were reviewed by 3 radiologists and one pulmonologist. On the other hand, the histology of lung tissues obtained by surgery or autopsy were examined by 4 pulmonological pathologists. Furthermore, the content of asbestos bodies in the lung was counted by phase-contrast microscopy. Thirteen cases were definitely diagnosed of asbestosis in the image including HRCT and 17 cases were diagnosed by the histopathological examination showing lung fibrosis with peribronchiolar fibrosis. Only 10 cases were indicated asbestosis by both the radiological and histopathological examinations. The mean value of asbestos bodies for these cases, was 2,133,255 per gram of dry lung tissue.

## 1. Introduction

In the diagnosis of asbestosis, it is considered to be most important to know about the presence of asbestos dust exposure in the occupational history, but it is not always easy to distinguish asbestosis from chronic interstitial pneumonia or other pneumoconiosis cases. In regard to the asbestosis cases diagnosed with the ILO International classification of Radiographs of pneumoconiosis (profusion rate (PR) of 1/1) or higher, we investigated the number of intrapulmonary asbestos bodies in addition to the occupational history, chest radiological findings, and pathological findings. Previously, while receiving cases as asbestosis in the ILO International classification of Radiographs of pneumoconiosis, we reported that there were cases in which asbestosis could not be diagnosed based on radiological or pathological/histological findings [1]

This time, for a total of 38 surgical and autopsy lung cancer cases that were diagnosed as asbestosis, we added clinical, radiological, and pathological investigations and report on definitive diagnoses of asbestosis from a comprehensive viewpoint.

## 2. Experimental Section

We targeted 38 cases diagnosed as asbestosis {PR 1/0 or higher} with the ILO International classification of Radiographs of pneumoconiosis in which lung parenchyma tissue was obtained through surgery or autopsy. Among the target cases 3 (7.9%) were asbestosis with lung cancer cases that underwent surgery and 35 (92.1%) were autopsy cases. There were 17 cases (44.7%) of death due to respiratory failure from asbestosis, 20 cases (52.6%) of asbestosis complicated with lung cancer, and 1 case (2.7%) of asbestosis complicated with pleural mesothelioma.

We examined characteristics such as gender, age, asbestos exposure in the occupational history, period of asbestos exposure, and clinical data in the pneumoconiosis management section PR classification. Since we needed to judge the presence of mixed dust pneumoconiosis, because workers in the construction and dismantling industries are often faced with instances in which they may inhale multiple types of inorganic substances, we carefully performed interviews and obtained pathological results [2].

In regard to the image findings for asbestosis, a representative system consisting of three respiratory radiologists and a pulmonologist consulted on the results of the chest x-ray and CT (including HRCT) imaging for the diagnosis. Furthermore, four respiratory pathologists performed histopathological diagnosis. We investigated whether or not asbestosis was present based on radiological or histopathological results, and selected cases that could be definitively diagnosed as asbestosis based on radiological and pathological results. The characteristics of the radiology that show asbestosis are defined as a fibrous change directly underneath the pleura such as subpleural dots, subpleural curvilinear lines, branching opacities, interlobular septum hyperplasia, *etc.* based on HRCT [3] and there are few images showing typical honeycomb lung and tractional bronchiectasis [4]. The presence of pleural plaque is a good indicator of asbestos exposure, but since pleural plaque is present even in cases of low exposure, this investigation withheld it as a reference observation [5]. Furthermore, pathological characteristics of asbestosis are centrilobular fibrosis developing at the periphery, and fibroblastic foci characteristic of chronic interstitial pneumonia are not often observed. More than 2 asbestos bodies/cm^2^ of lung tissues are observed by light microscopy. Based on these criteria pathological discrimination from other disease was made [6].

Lung parenchyma tissue that is free of carcinomatous infiltration, acute pneumonia, *etc.* is dissolved based on the Kohyama method [7], and the number of asbestos bodies is estimated per gram of dry weight lung tissue. It was reported that for asbestosis to develop a level of asbestos exposure exceeding 25 fibers/mL of air X year is required [8], and we judged that less than 5,000 bodies/g dry weight lung tissue indicated a low probability of asbestosis.

## 3. Results

Among the target cases, there were 37 male cases and only 1 female case. In regard to the age distribution, the largest group was 70 or younger consisting of 17 cases (44.7%), the majority was 71 or older, and the average was 71.6 ± 9.3 years (median age was 72 years). In terms of the occupational history, the largest group consisted of 19 people (50%) who worked in the dockyards and among them those working with the rigging of ships represented a majority of 10 cases (52.6%) who were exposed to comparatively high concentrations of asbestos. On the other hand, there were a total of 8 cases (Table 1) from other occupations exposed to high concentrations of asbestos: 5 cases of spraying of asbestos, 2 cases of insulation work, and 1 case of asbestos product manufacturing work.

There were 31 cases (81.6%) representing a majority who were exposed to asbestos for over 20 years, and the average was 30.3 ± 12.52 years (mean value of 32.5 years). In the previously mentioned 8 cases who were exposed to high concentrations of asbestos in their work, 5 cases who were involved with asbestos spraying work were exposed for a relatively short number of years of 7–22.2 years (mean value of 18 years). There were 5 cases that did not indicate pneumoconiosis findings of PR1/1 classification or greater at the time of diagnosis. These 5 cases, although the chest CT showed fibrosis indicating asbestosis, the chest x-ray findings indicated a classification of PR1/1. Furthermore, there were 11 cases categorized as PR1, 13 cases categorized as PR2, and 9 cases categorized as PR3. The majority of the cases were classified as PR2 or higher.

There were 13 cases in which asbestosis characteristics which included subpleural dots, curvilinear lines, branching opacities etc show the centrilobular fibrosis were manifested in the images including High Resolution CT (HRCT). There were 4 cases that showed subpleural dots, subpleural curvilinear lines, branching opacities, and interlobular septum hyperplasia [3] in the HRCT which indicated asbestosis. These 17 cases (44.7%) of diagnosed asbestosis were based on radiology. The PR classifications for the 17 cases were 2 cases of PR1, 10 cases of PR2, and 5 cases of PR3.

**Figure 1.**
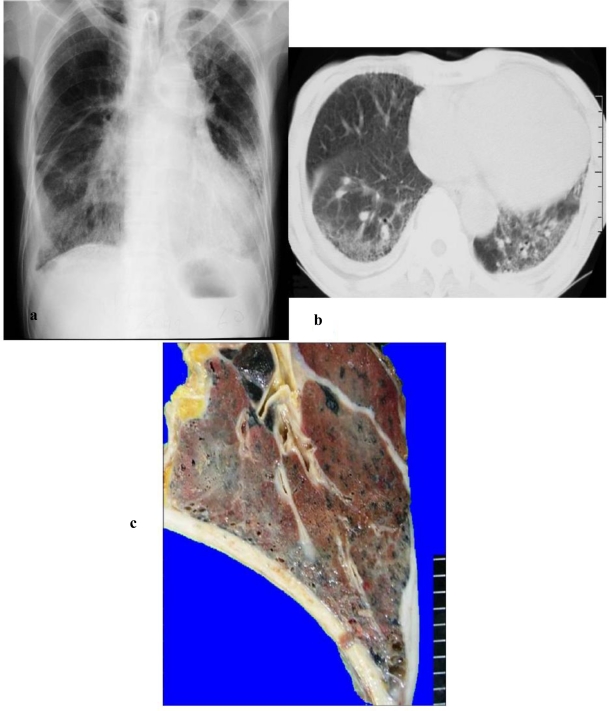

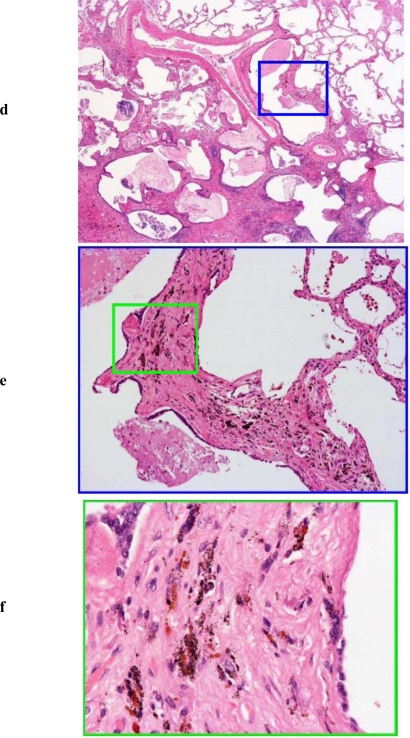
This case was diagnosed as asbestosis based on chest x-ray, CT, and pathology. Radiological findings showed characteristics of ground glass shadows in both lower lungs accompanied by bilateral pleural thickening (a). Chest CT showed slight honeycombing of the lungs but mainly ground glass shadows (b). On the other hand, visual inspection of autopsied lungs indicated a few small honeycomb lungs and they were atypical (c). Histopathological findings showed fibrosis accompanied by a large number of asbestos bodies on the respiratory bronchiole wall and the surrounding area and severe fibrosis accompanied by the honeycomb lungs (d, e, f). There were more than 2,280,000 asbestos bodies/g in the lung.

**Figure 2.**
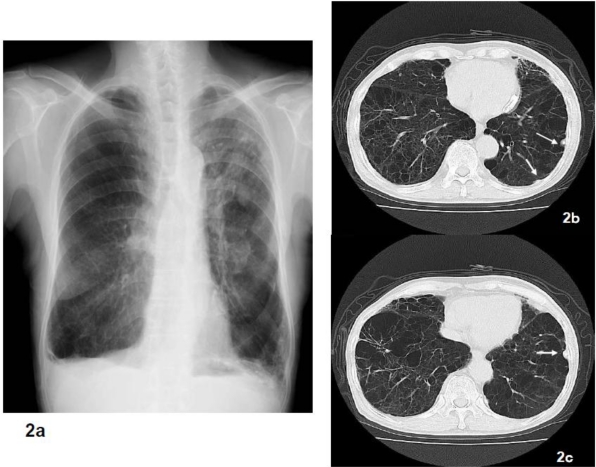
Although pulmonary emphysema was indicated by radiologically in this case, the pathological findings were characteristic of asbestosis. Pulmonary emphysema was diagnosed based on the chest x-ray (2a), chest CT (2b, 2c) indicated fibrosis accompanied by pulmonary emphysema. However, there were 668,447 asbestos bodies/g in the lungs and histopathologically there were findings of fibrosis of the bronchiole wall and surrounding area accompanying the asbestos bodies.

On the other hand, there were 11 cases in which there were fibrosis findings in the chest x-ray, and it was judged there was the possibility of asbestosis in these cases. Furthermore, there were 6 cases diagnosed with classical asbestosis (Figure 1). There was only one case of atelectasis hardening which is the most typical type of asbestosis. However, there were 6 cases in which there were only findings of pulmonary emphysema (Figure 2) or where the presence of fibrosis could not be clarified based on chest x-ray.

In terms of pleural lesions, there were 30 cases (78.9%) with medical findings of asbestos exposure such as pleural plaque, and only 4 cases of diffuse pleural thickening were found. Namely, the presence of pleural plaque could not be confirmed in 8 cases. Among the 17 cases of diagnosed asbestosis based on radiology, there were 3 cases (17.3%) in which pleural plaque was not confirmed.

**Figure 3.**
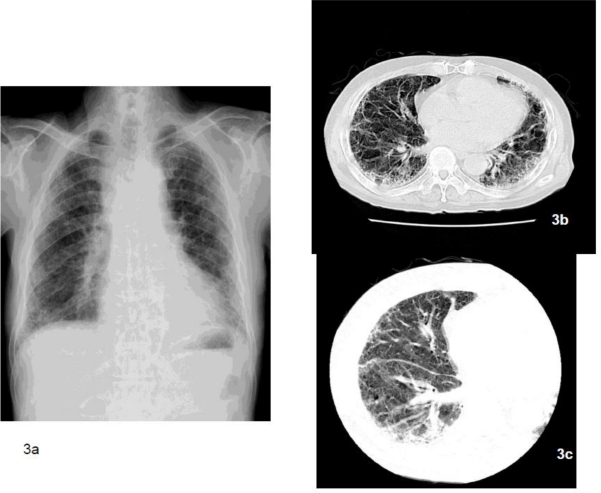
The chest x-ray (3a) indicates bilateral irregular opacity, and asbestosis was also diagnosed based on occupational asbestos exposure. However, pathologically we could not confirm the diagnosis of asbestosis in this case. The main radiological findings indicated that ground glass shadows (3b, 3c) did not accompany the honeycomb lungs. The fibrosis was observed parallel to the bronchovascular bundle. These were not inconsistent with the diagnosis of asbestosis. However, findings of fibrosis starting from the surrounding area of the bronchioles, which characteristically is the beginning of asbestosis based on pathology, were scarce and evidence of honeycomb lungs was not apparent. Asbestos bodies were sparse inside the lungs and there were 7,482 asbestos bodies/g of dry lung tissue.

**Figure 4.**
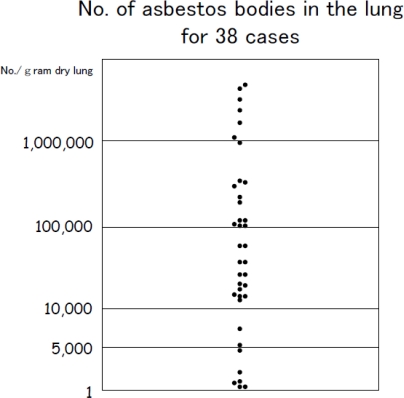
Number of asbestos bodies in the lung for the targeted 38 asbestosis cases. The figure shows the large difference among the cases from the fewest of 300 bodies/g of dry lung tissue to the most of 2,780,000 bodies/g of dry lung tissue.

There were 17 cases (44.7%) that indicated histopathologically bronchial wall fibrosis, peripheral fibrosis, or fibrosis that was non-contradictory to asbestosis. Although pathologically it is characteristic of asbestosis that asbestos bodies are present on the bronchial wall or there is peripheral fibrosis, there were 3 cases (7.9%) in which chest x-ray did not indicate fibrosis. Furthermore, there were 21 cases of honeycomb lungs and almost the same number of cases (17 cases) without it (Table 2). There were also 12 cases (Figure 3) in which asbestos bodies were not present in histopathological specimens (Table 2). Among all of these cases, there were only 10 cases (26.3%) in which both the radiological and histopathological examinations indicated asbestosis (Table 3). In the occupational histories, there were 5 cases of asbestos spraying work, 4 cases of dockyard rigging work, and 1 case of asbestos product manufacturing work (Table 3). Furthermore, these 10 cases were exposed to extremely high concentrations of asbestos in which the average concentration of asbestos bodies in the lung was 1,434,594 ± 901,861 (mean value of 1,379,827) (Figure 4). The pneumoconiosis classification for these cases was 1 case of PR1/0, 1 case of PR1/1, 3 cases of PR2/2, 1 case of PR2/3, 3 cases of PR3/2, and 1 case of PR3/3.

**Figure 5.**
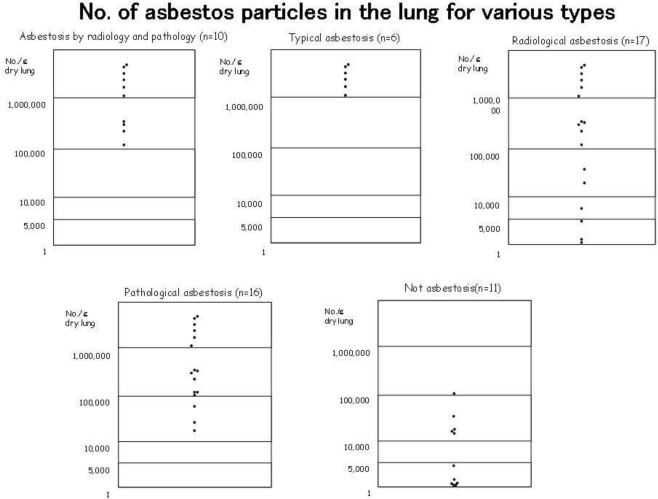
The number of asbestos bodies in the lung for the 10 cases where the clinical and radiological diagnoses matched the pathological diagnosis for asbestosis; the 6 cases where the clinical, radiological, and pathologically findings showed typical asbestos; the 17 cases of asbestosis diagnosed based on clinical findings and radiology; and 16 cases of pathologically diagnosed asbestosis. In the case of typical asbestosis, all 6 cases had more than 1,000,000 bodies. However, among the 17 asbestosis cases diagnosed based on clinical and radiological findings, 3 cases had less than 5,000 bodies.

The concentration of asbestos bodies in the lung for the cases where asbestosis could be diagnosed based on radiology was the average of 873,978 ± 966,829 (mean value of 451,323) and that for cases where asbestosis could be diagnosed based on pathology was the average of 965,387 ± 945,259 (mean value of 657,727). For the 6 cases in which we find typical asbestosis based on radiological and histopathological findings there were the average of 2,068,255 ± 568,089 bodies (mean value of 2,133,255) and all cases exceed 1,000,000 bodies/g of dry lung tissue (Figure 5). There were also 6 cases in which there were 5,000 bodies or less. In the occupational histories of these 6 cases, there were 2 cases each in which they worked in construction and ironworks, and there was 1 case each in which the patient worked in brick production and hoisting (crane) work in a dockyard. In these cases, based on radiology and pathology they were diagnosed not as asbestosis, but instead as emphysema accompanied by fibrosis.

As above, although pathologically asbestosis is indicated, in 5 cases based on radiology we did not find more than 1 type of results for asbestosis and 6 other cases were thought not to be asbestosis when taking into account all radiological results, pathological findings, asbestos particle concentration, and occupational history. For the total of these 11 cases, we conclude that comprehensively that these were not asbestosis and other 4 cases were possible asbestosis.

## 4. Discussion

The asbestosis guidelines published by the American Thoracic Society (ATS) [9]. in 2004 state that (1) pathological changes in asbestos related diseases shown in radiological and pathological results agree with morphological findings, (2) findings suggesting asbestos inhalation such as pleural plaque and asbestos exposure in the occupational history and asbestos particle detection on the basis of asbestos inhalation, and (3) discrimination from other diseases that are the cause of morphological abnormalities are all reasons for judging asbestosis. However, there are cases in which it is not always easy to make a diagnosis using only this guideline. We previously reported on an investigation targeting 25 asbestosis cases in which 6 cases based on clinical, radiological, histopathological, and comprehensive results were concluded that they could not be diagnosed as asbestosis [1].

In this investigation we re-examined the 25 cases based on radiological or histopathological results and added 13 new cases. In terms of gender there were 37 male cases and 1 female case who was involved in rigging in a dockyard. A majority of the cases were 71 years or older and the mean value was 72 years. In terms of the period of occupational exposure to asbestos, the mean value is 32.5 years and many were exposed for relatively long periods during their work. There were 5 cases in which their work involved asbestos inhalation for the period of 7–22 years, and they were exposed for short periods but at high concentrations.

In addition, based on the PR classification in the pneumoconiosis method, there were 5 cases classified as PR0/1 that were unable to be diagnosed as asbestosis. On the other hand, there were 9 cases classified as PR3 and the majority of the completed asbestosis cases were classified as PR2 or higher. Six of 13 cases (46.2%) were typical asbestosis cases, and were diagnosed based on clinical, radiological, and pathological results. Furthermore, we confirmed extremely high exposure levels in all of these 6 cases where the number of asbestos bodies in the lung exceeded 1,000,000 bodies/g of dried lung tissue. The occupations of 4 people involved asbestos spraying, and these cases showed classical pathological images. One case of atelectasis hardening and one case of asbestos product manufacturing were diagnosed with classic asbestosis based on clinical and pathological results.

On the other hand, there were 17 cases that could be diagnosed based only on radiology. However, 11 cases except for the 6 cases where the number of asbestos bodies exceeded 1,000,000 bodies showed, based on chest CT (including HRCT), centrilobular fibrosis indicating subpleural dots and subpleural curvilinear lines, *etc.* [3], while they did not show, as a cardinal symptom, a typical honeycombing or tractional bronchiectasis suggesting IPF/UIP. Based on these considerations, we concluded the diagnosis of asbestosis. Among these cases, there were 3 cases (17.6%) in which pleural plaque was not observed. Although pleural plaque is an indicator of asbestos exposure, even low-level exposure can yield pleural plaque, and the presence of fibrosis lesions do not necessarily lead to the diagnosis of asbestosis. However, from these 11 cases diagnosed by radiological findings in chest CT, only 4 cases were diagnosed with asbestosis based on pathological findings. In the other 7 cases no fibrosis was found around the bronchioles which would indicate pathological findings to diagnose asbestosis. Instead we found mainly honeycombing of the lungs, and we could not definitively determine that the cause was asbestos exposure. Fibrosis from asbestosis is caused by the depositing of asbestos fibers in respiratory bronchioles that cause irritation, then respiratory fibrosis begins and it progresses to the surrounding tissue [10]. On the other hand, in chronic interstitial pneumonia since small air spaces become clogged in the respiratory tract it is judged pathologically that fibrosis begins from the most remote location. However, once fibrosis progressed to a honeycomb lung, we cannot judge if it is asbestosis or chronic interstitial pneumonia. For this reason, except for the point regarding whether or not asbestos bodies exist, in the pathological diagnosis of completed asbestosis, it is difficult to judge that it is another type of interstitial pneumonia.

There were 16 cases in which the diagnosis was asbestosis based on pathological findings, 6 of theses cases were classical asbestosis, and 4 other cases had characteristics of asbestosis based on radiological results. The PR classifications for the cases of histopathology based asbestosis were 3 cases of type 0, 4 cases of PR1, 4 cases of PR2, and 7 cases of PR3. Four of the remaining 6 cases indicated pathologically confirmed fibrosis from the bronchiole wall or the surrounding area but showed only minor findings based on radiology, and the chest x-ray could not confirm fibrosis of classification PR1/1 or higher. Furthermore, there were two cases in which findings of pulmonary emphysema were the main indication but there were only minor findings of fibrosis. More specifically, even though they showed histopathological findings of asbestosis of Grade I–II, or Grade III, their chest x-rays showed only a minor level of fibrosis that does not exceed the PR1/1 classification of an irregular shaped shadow. In these cases, although subpleural dots, interlobular septum hyperplasia, *etc.* are detected at a comparatively early stage in HRCT imaging when looking for asbestosis, asbestosis with classification PR1/0 or higher could not be diagnosed using the pneumoconiosis method. On the other hand, among the 38 cases there were 12 cases (31.6%) in which asbestos bodies were not observed in lung tissue specimens, and these cases did not conform to the Helsinki criteria [11] of more than 2 bodies/cm^2^ in the lung tissue, which is the pathological diagnosis standard for asbestosis. More specifically, even if there is an occupational history indicating asbestos exposure and agreement in the findings of asbestosis based on chest x-ray and CT, we found that there are cases in which asbestosis cannot be diagnosed based on pathological results.

Even though there was agreement on the pathological results of asbestosis, all the cases in which diagnosis of asbestosis could not be made based on radiology with a classification of PR 1/0 or higher using the pneumoconiosis method were confirmed in this study to have calcified pleural plaque. For this reason, we have findings of pleural plaque with irregular shaped shadows based on chest x-ray and confused diagnosis of asbestosis with the classification of PR1/1 or higher.

There was no major distinction in the number of asbestos bodies in the lung for cases diagnosed with asbestosis based on radiology, 873,978 ± 966,829 (mean value of 451,32), compared to that for the cases diagnosed with asbestosis based on histopathological results, 965,387 ± 945,259 (mean value of 657,727). On the other hand, the number of the asbestos bodies in the lung for cases of asbestosis diagnosed based on comprehensive investigation including clinical, radiology, and pathological results was extremely large, 1,434,594 ± 901,861 (mean value of 1,379,877), and this suggested that unless the patient was not exposed to an exceedingly high concentration of asbestos, typical asbestosis would not manifest. On the other hand, it was reported that for asbestosis to develop a level of asbestos exposure exceeding 25 fibers/mL of air X year is required. In this investigation, there were 6 cases in which the level did not reach 5,000 bodies, and were not subjected to diagnosis. All of these 6 cases were not exposed to high levels of asbestos, and due to this, they were not diagnosed with asbestosis which did cause any inconsistency in the cases. However, for chrysotile inhalation, we cannot always detect more than 5,000 asbestos bodies in the lung, because chrysotile does not easily form asbestos bodies. Therefore the types and numbers of asbestos fibers should be determined.

This investigation targeted cases that were clinically diagnosed with asbestosis and received pneumoconiosis management section classification. These cases include those resembling pneumoconiosis in which the radiology showed asbestos dust contained in other dust that was inhaled. The diagnosis of asbestosis does not always require pathological findings. If we focus mainly on the radiological findings of asbestos exposure, the occupational history becomes important [12,13]. However, since work environments in which workers are exposed to high concentrations of asbestos are almost all gone in Japan, in the future we will need to perform investigations to conclude a diagnosis of actual asbestosis.

As mentioned above, we established that in order to diagnose asbestosis asbestos exposure in the occupational history and the existence of pleural plaque as an asbestos exposure indicator are important, but to reach a definitive diagnosis detailed radiological findings, and if necessary pathological findings, are useful. In this investigation, we focused on autopsy cases, but in the future we hope to investigate more extensively cases including those after lung cancer surgery and on a larger scale.

## 5. Conclusions

The diagnosis of radiological asbestosis is difficult for the differential diagnosis from IPF/UIP or mixed dust pneumoconiosis. And the discrepancy for the diagnosis of the radiological and histopathological examination is problem for the diagnosis of asbestosis.

## Figures and Tables

**Table 1.** Occupational history.

**Occupation**	**No.**
Dockyards	19
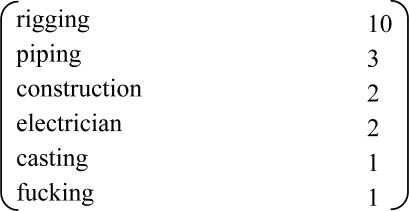	
Spraying asbestos	5
Insulating	2
Construction	2
Iron working	2
Repairing boiler	2
Repairing furnace	1
Mixing asbestos and asphalt	1
Dismantling	1
Asbestos products maker	1
Making bricks	1
Furnishing	1

**Table 2.** Honey combing and asbestos bodies in the histology.

	No. of cases
Honey combing	Yes 21
	No 17

Asbestos body	
0 body	12
0< <2 bodies	6
2 bodies≤ <20 bodies	13
20 bodies ≥	7

**Table 3.** Cases of asbestosis by radiological and pathological findings.

	**occupational histories**	**exp. Term**	**gender**	**age**	**cause of death**	**PR**	**No. of bodies**
(1)	Spraying asbestos	18 y	M	63	Resp.failure	1/1	2,650,000
(2)	Spraying asbestos	12 y	M	58	Resp.failure	2/2	1,634,726
(3)	Spraying asbestos	22 y	M	48	Resp.failure	2/2	2,733,078
(4)	Spraying asbestos	7 y	M	60	Resp.failure	2/3	1,946,837
(5)	Rigging	24 y	M	72	Resp.failure	3/2	647,007
(6)	Rigging	41 y	M	65	Lung cancer	3/3	156,151
(7)	Rigging	30 y	M	70	Resp.failure	3/2	451,323
(8)	Rigging	40 y	F	85	Lung cancer	2/2	1,124,918
(9)	Furnishing	34 y	M	61	Resp.failure	1/0	681,933
(10)	Asbestos maker	22 y	M	68	Resp.failure	3/2	2,319,969

## Abbreviations

PR: Profusion rates
HRCT: High resolution CT
IPF/UIP: interstitial pulmonary fibrosis/usual interstitial pneumonia
